# The Outcome of Patients with Mild Stroke Improves after Treatment with Systemic Thrombolysis

**DOI:** 10.1371/journal.pone.0059420

**Published:** 2013-03-19

**Authors:** Xabier Urra, Helena Ariño, Laura Llull, Sergio Amaro, Víctor Obach, Álvaro Cervera, Ángel Chamorro

**Affiliations:** 1 Functional Unit of Cerebrovascular Diseases, Hospital Clínic, Barcelona, Spain; 2 Institut d’Investigacions Biomediques August Pi i Sunyer, Barcelona, Spain; 3 Medicine Department, School of Medicine, Universitat de Barcelona, Barcelona, Spain; Charité Universitaetsmedizin Berlin, Germany

## Abstract

**Introduction:**

In up to one third of patients with mild stroke suitable to receive systemic thrombolysis the treatment is not administered because the treating physicians estimate a good spontaneous recovery. However, it is not settled whether the fate of these patients is equivalent to those who are thrombolysed.

**Methods:**

We analyzed 203 consecutive patients (134 men and 69 women, mean age 69±14 years) without premorbid disability and a NIHSS score ≤5 at admission [median 3 (IQR 2–4)]. Intravenous thrombolysis was administered within 4.5 hours from stroke onset (n = 119), or it was withheld (n = 84) whenever the treating physician predicted a spontaneous recovery. The baseline risk factors, clinical course, infarction volume, bleeding complications, and functional outcome at 3 months were analyzed and declared to a Web-based registry which was accessible to the local Health Authorities.

**Results:**

Expectedly, not thrombolysed patients had the mildest strokes at admission [median 2 (IQR 1–3.75)]. At day 2 to 5, the infarct volume on DWI-MRI was similar in both groups. There were no symptomatic cerebral bleedings in the study. An ordinal regression model adjusted for baseline stroke severity showed that thrombolysis was associated with a greater proportion of patients who shifted down on the modified Rankin Scale score at 3 months (OR 2.66; 95% CI 1.49–4.74, p = 0.001).

**Conclusions:**

Intravenous thrombolysis seems to be safe in patients with mild stroke and may be associated with improved outcome compared with untreated patients. These results support the evaluation of the efficacy of intravenous thrombolysis in mild stroke patients in randomized clinical trials.

## Introduction

Current guidelines offer no specific recommendations on the need of thrombolysis in stroke patients with mild or rapidly improving symptoms [1], [2]. However, this issue is of great clinical interest as up to half of ischemic stroke patients manifest mild or rapidly improving symptoms at clinical onset [3], [4], and around 30% of these patients are not treated with thrombolytic agents on the assumption that they may achieve an excellent recovery spontaneously [5]–[7]. However, according to some reports [5], [6], [8]–[11], up to one third of these patients fail to recover as much as it was anticipated by the responsible physician, and persist having symptoms as the result from a delayed growth of the infarction.

The criteria used to define mild or rapidly improving symptoms are also weak and may encounter rather loose terms such as isolated sensory loss, ataxia, facial weakness, or dysarthria [12]. In most research studies, clinical deficits measured on the National Institutes of Health Stroke Scale (NIHSS) score of up to 3 or 5 were the most common definitions of mild stroke [13].

We analyzed the clinical and radiological course of patients with mild or rapidly improving stroke on admission in which the decision to administer intravenous recombinant tissue plasminogen activator (IV rt-PA) within 4.5 h of stroke onset was judged by the responsible physician according to his prediction of spontaneous recovery.

## Methods

### Ethics Statement

The study protocol was approved by the Clinical Research Ethics Committee of the Hospital Clínic de Barcelona (CEIC Hospital Clínic) and the patients or their legal representatives signed a written informed consent if age was over 80 years, or treatment was to be initiated >3 h of stroke onset.

### Patients

All consecutive patients with ischemic stroke admitted at our institution from January 2009 to May 2012 which fulfilled all the following criteria: 1/delay from the onset of symptoms to treatment less than 4.5 h; 2/admission or post-imaging NIHSS score ≤5; 3/premorbid modified Rankin Scale (mRS) ≤1; and 4/no absolute contraindications to IV rt-PA.

At our center, the decision to administer IV rt-PA in patients with mild strokes depends on the estimated probability of achieving a full recovery spontaneously. Accordingly, patients with very low NIHSS scores on admission are less likely to receive IV rt-PA than patients with higher NIHSS scores.

All the study participants were admitted at an intermediate care Stroke Unit and managed by certified stroke neurologists following the European Stroke Organization Guidelines [2]. The NIHSS score was assessed at hospital arrival and after baseline brain imaging (pretreatment score), at day 1, at day 7 or discharge, and at day 90. The functional outcome was assessed with the mRS at day 90 in patients that pertained to the geographical area of coverage of the institution (450.000 inhabitants). In the remainder, the mRS at hospital discharge was carried forward and used in the primary efficacy analysis. Descriptive statistics were performed to evaluate the similarities between the patients assessed at discharge and/or at 3 months. The qualifying symptoms were classified according to the Oxfordshire Stroke Project Classification (OCSP) and the stroke etiology was classified according to the Trial of Org 10172 in Acute Stroke Treatment (TOAST) criteria after a complete diagnostic workup [14]. In addition to a baseline CT scan all patients had a head CT scan within 24 h of admission in which hemorrhagic conversion was graded according to ECASS criteria as hemorrhagic infarction (HI) 1 and 2, parenchymal hemorrhage (PH) 1 and 2, and remote PH (PHr) 1 and 2 [15]. Symptomatic intracranial hemorrhage (sICH) was defined as any bleeding associated with an increment of at least 4 points in the NIHSS score. The volume of infarction was assessed on DWI-MRI between day 2 and 5 of stroke onset using AMIRA software (Visage Imaging, Inc., San Diego, CA). The brain infarctions were further categorized as lacunar, territorial or watershed [16].

Data were prospectively collected in a local database that included demographics, risk factors, main laboratory results, neuroimaging, concomitant therapies, clinical course, and functional outcome. In addition, data were declared to a Web-based registry that satisfied all legal requirements for protection of personal data and was monitored by the Catalan Health Department [17].

### Statistics

Normal distribution of all studied variables was assessed, and continuous variables were compared with Student’s t-test, ANOVA, Mann-Whitney or Kruskal-Wallis tests as appropriate. Correlations were assessed with Spearman coefficients, and categorical variables were compared with the Fisher’s exact tests. Ordinal regression analysis was used to increase the statistical power of the study [18], and analyze the independent effect of thrombolytic therapy on functional outcome at 3 months. As it was anticipated that patients not treated with thrombolysis would have the mildest strokes, the pretreatment NIHSS score was forced *a priori* into the model. In addition, exploratory analyses were also performed and which included the variables associated to outcome (p<0.2) on the univariate analysis and the variables with significant differences between treatment groups at baseline. The level of significance was established at a two-tailed value of p<0.05. All tests were performed using SPSS version 20.0.

## Results

During the 40 months of the study, 2560 acute stroke patients were assessed including 875 patients admitted within 4.5 h of clinical onset. Of those, 313 patients had a NIHSS score <5 on admission but 110 patients were not included in the study as they had a pre-morbid mRS >1 (n = 91), absolute contraindications for IV rt-PA (n = 13) or received endovascular therapy (n = 6). Therefore, the study included 203 patients (8% of the total population) that received IV rt-PA (119 patients), or standard care only (n = 84). As previously anticipated, the thrombolysed patients had a greater NIHSS score than the untreated patients at baseline (Table 1).

**Table 1:** General characteristics of the study population according to the treatment group.

	Thrombolysis (n = 119)	No thrombolysis (n = 84)	P
Age, years, mean (SD)	68.8 (13.8)	69.0 (13.2)	0.921
Gender, %, male/female	68.9/31.1	61.9/38.1	0.300
Hypertension, %	68.1	67.5	0.929
Diabetes mellitus, %	24.4	37.3	0.047
Dyslipidemia, %	38.8	48.2	0.186
Coronary artery disease, %	16.9	18.1	0.836
Atrial fibrillation, %	15.3	23.8	0.115
Prior mRS, median (IQR)	0 (0–0)	0 (0–1)	<0.001
Onset to admission time, min, median (IQR)	94 (58–143)	108 (60–171)	0.198
SBP, mmHg, mean (SD)	157.5 (24.5)	163.1 (33.3)	0.221
Glucose, mg/dL, mean (SD)	138.3 (54.8)	141.7 (59.4)	0.683
OCSP, %, Lacunar/Non-lacunar	37.8/62.2	30.8/69.2	0.316
**NIHSS score, median (IQR)**			
Admission	3 (2–4)	2 (1–3.75)	<0.001
Pre-treatment/post neuroimaging	3 (2–4)	1 (0–2)	<0.001
Day 1	1 (0–3)	0 (0–1)	<0.001
Discharge	1 (0–2)	0 (0–1)	0.027
Day 90	0 (0–1)	0 (0–0.75)	0.926
Improvement in NIHSS during admission, median (IQR)	1 (0–3)	0 (0–1)	<0.001
DWI volume, median (IQR), n = 152, cc	0.69 (0–3.09)	0.33 (0–2.46)	0.165
DWI lesion pattern, %			0.417
Territorial	50.5	50.0	
Lacunar	20.4	14.3	
Watershed	2.2	0	
No lesion	26.9	35.7	
TOAST			0.566
Cardioembolic, %	28.4	28.4	
Large vessel, %	14.7	6.8	
Lacunar, %	20.2	23.0	
Undetermined, %	34.9	39.2	
Other, %	1.8	2.7	
Stroke mimick, %	6.8	8.6	0.637
Any ICH, %	6.7	2.7	0.323
sICH, %	0	0	
mRS at discharge, median (IQR)	1 (0–2)	1 (1–2)	0.966
mRS at day 90, median (IQR)	1 (0–1)	1 (0–1)	0.023
Death, %	1.7	3.6	0.651

mRS: modified Rankin scale; SBP: systolic blood pressure, OCSP: Oxfordshire Stroke Project Classification; TOAST: Trial of Org 10172 in Acute Stroke Treatment; NIHSS: National Institutes of Health Stroke Scale; ICH: intracranial hemorrhage; sICH: symptomatic intracranial hemorrhage.

The patients treated with IV rt-PA showed higher NIHSS scores at day 1, and at day 7 or discharge, but these differences did not persist at day 90 (Table 1). In 149 patients in whom a DWI-MRI was performed between day 2 and 5, the volume of infarction did not differ between patients allocated to IV rt-PA or to standard care (Table 1). The pattern of infarctions observed in order of occurrence on these patients included territorial infarcts (50%), normal brain MRIs (30%), lacunar infarctions (18%), and watershed infarctions (1%). As shown in Table 1 there were more patients with a normal MRI in patients receiving standard care only, but the differences were not significant. The overall rate of hemorrhagic complications in the study population was very low, including 5.2% of asymptomatic ICH, and 0% sICHs (95% CI 0–3.13% for treated patients and 0–4.37% for not treated patients). There were two cases of HI1 in non-thrombolysed patients and two cases of PH1, one rPHr1, one HI2 and four HI1 cases in thrombolysed patients.

### Outcome

**Figure 1:**
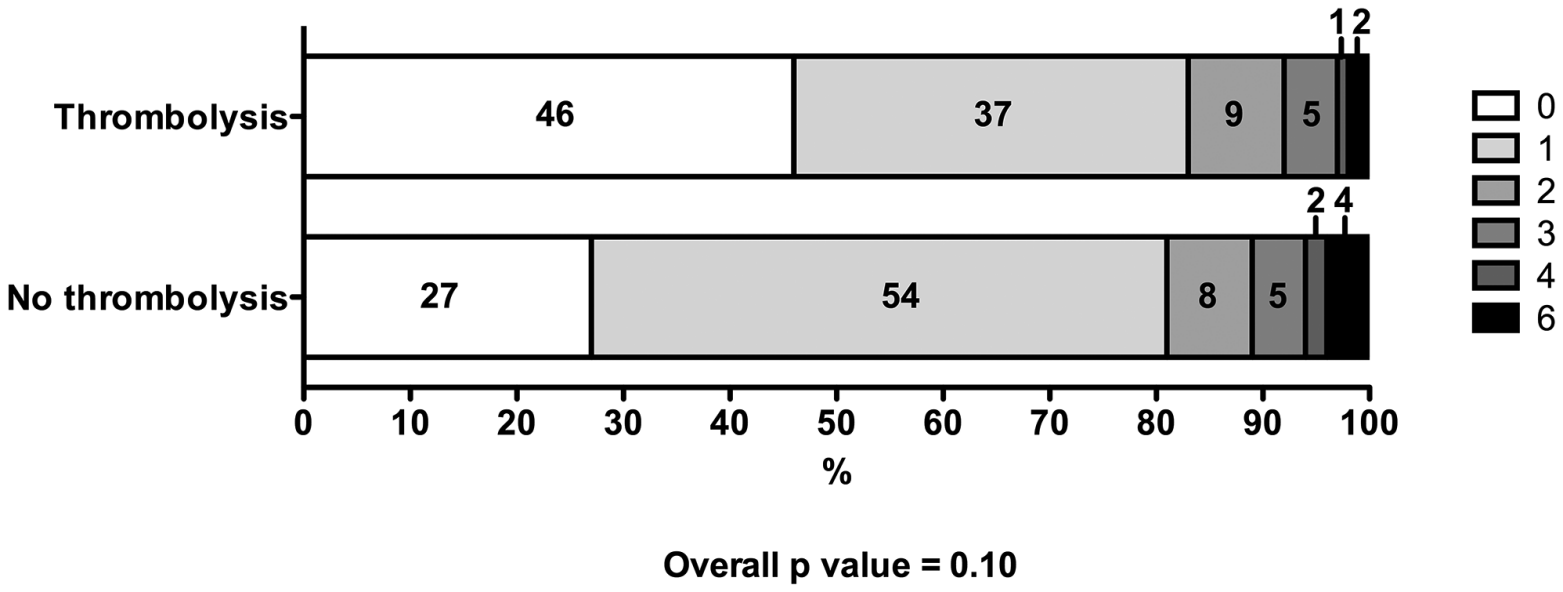
Distribution of modified Rankin Scales scores at 3 months in patients with mild stroke and treated or not with intravenous thrombolytic therapy.

In the study, 53 of 203 patients referred from other centers were assessed only at hospital discharge (27 treated with thrombolysis and 26 not treated, p = 0.247). The baseline traits of these patients did not differ from the patients with 3 months follow up (data not shown). At 3 months, 167 (82%) patients had excellent outcome (mRS 0 to 1). As shown in Figure 1, there were no significant differences in the proportion of patients that reached excellent outcome (83% in patients that received IV rt-PA versus 81% in patients that received standard care).

In the ordinal regression analysis, we found a greater proportion of patients who shifted down on the modified Rankin Scale score at 3 months in the thrombolysed group than in patients receiving standard care (OR 2.66; 95% CI 1.49–4.74, p = 0.001). The association also was significant (OR 2.02; 95% CI 1.02–3.98; p = 0.042) in models adjusted for the effect of all variables associated to mRS score at 3 months on univariate comparison and for variables with differences at baseline: pretreatment NIHSS (OR 0.77; 95% CI 0.67–0.89; p<0.001), age (OR 0.97; 95% CI 0.95–1.00; p = 0.023), prior mRS (OR 0.35; 95% CI 0.17–0.70; p = 0.003), dyslipidemia (OR 0.62; 95% CI 0.33–1.20; p = 0.157), coronary artery disease (OR 0.50; 95% CI 0.24–1.05; p = 0.068), hypertension (OR 0.78; 95% CI 0.40–1.52; p = 0.468), diabetes (OR 0.64; 95% CI 0.30–1.36; p = 0.243), systolic blood pressure (OR 0.99; 95% CI 0.98–1.00; p = 0.128) and glucose levels (OR 1.00; 95% CI 0.99–1.01; p = 0.625). In exploratory analysis, there was also a trend in the same direction for thrombolysis (OR 1.85; 95% CI 0.90–3.86; p = 0.100) after excluding patients without day 90 visit.

## Discussion

The value of systemic thrombolysis in patients with mild or rapidly improving stroke is not settled. A post-hoc analysis of very few patients with mild stroke assessed in the NINDS trial suggested some benefit [19], and a subgroup analysis of the IST-3 trial did not show a significant effect of rt-PA in patients with mild stroke [20]. This study confirmed that in regular practice a significant proportion of patients with mild stroke are not deemed suitable to be thrombolysed. However, the major new finding of the study was that the administration of IV rt-PA was independently associated with a greater proportion of patients shifting down on the modified Rankin Scale score at 3 months compared with patients receiving standard care. Importantly, the association was obtained regardless that the patients who did not receive IV rt-PA had greater chances of recovery as they were in better neurological condition at baseline [21], [22]. Indeed, this benign clinical course influenced the treating physician who withheld the therapy to avoid unnecessary risks in patients likable to make a full recovery spontaneously. Therefore, it is very likely that the true beneficial effects of IV rt-PA in patients with mild stroke might be even stronger.

The rate of excellent outcome was higher in this study than in previous reports of patients with minor stroke where the functional outcome was evaluated only at hospital discharge [8], [10], [11]. Most likely, the good results of the current study obeyed to the longer duration of follow up, the admission and management of all patients into a stroke dedicated unit, and the exclusion of patients with any degree of premorbid disability.

The study also highlighted the safety profile of IV rt-PA in patients with mild or rapidly improving stroke as none of the actively treated patients suffered symptomatic bleeding complications. Therefore, any future clinical trial designed to compare the value of rt-PA versus standard care in patients with mild stroke must calculate the inclusion of a very large database.

While the patients included in this study represented only 8% of all the stroke admissions at our Stroke Unit, they also represented 24% of all the ischemic stroke patients which are candidates to receive IV rt-PA within 4.5 h. Therefore, these results may have important clinical implications for many of these patients are currently not thrombolysed in regular practice on the assumption of their benign natural course. As the study showed, 19% of these patients failed to achieve a full recovery. Yet, while the study confirmed a high rate of excellent recovery in many of these patients, it also suggested that the benefits were enhanced after thrombolysis.

The main limitation of the study was its non-randomized design although several of its traits minimized the risk of bias. Thus, the effect of the lower stroke severity on the untreated group was minimized by the appropriate adjustment of the NIHSS score in multivariate analysis. The validity of the study was also supported by the prospective collection of the data and its storage into a Web-based registry owned and monitored by the Catalan Health authorities [17]. Therefore, our results represented all the admissions at our institution that fulfilled the preestablished entry criteria. Alternatively, most of previous cohorts described the outcome of either treated [23]–[25] or not treated patients [6]–[10], or used historical controls [26].

Currently, a randomized controlled clinical trial is being planned to evaluate the role of thrombolysis in patients with mild stroke [27]. In the meantime, our findings may be very informative for the treating physicians as they support a favorable benefit/risk ratio of thrombolysis in patients with minor stroke.

### Conclusions

Patients with mild or rapidly improving stroke seem to benefit from a timely administration of IV rt-PA. More patients receiving IV rt-PA shift down on the mRS at 3 months compared to patients receiving standard care. The risk of serious bleeding complications is very low in these patients while the probability of incomplete recovery is not negligible in untreated patients. While awaiting a definitive answer from a randomized clinical trial our results support the use of IV rt-PA in patients with mild or rapidly improving stroke when there are no contraindications.
